# Development of a prediction model on preeclampsia using machine learning-based method: a retrospective cohort study in China

**DOI:** 10.3389/fphys.2022.896969

**Published:** 2022-08-12

**Authors:** Mengyuan Liu, Xiaofeng Yang, Guolu Chen, Yuzhen Ding, Meiting Shi, Lu Sun, Zhengrui Huang, Jia Liu, Tong Liu, Ruiling Yan, Ruiman Li

**Affiliations:** ^1^ The First Affiliated Hospital of Jinan University, Guangzhou, China; ^2^ School of Information and Communication Engineering, Harbin Engineering University, Harbin, China

**Keywords:** preeclampsia, machine learning, prediction, deep neural network, pregnancy

## Abstract

**Objective:** The aim of this study was to use machine learning methods to analyze all available clinical and laboratory data obtained during prenatal screening in early pregnancy to develop predictive models in preeclampsia (PE).

**Material and Methods:** Data were collected by retrospective medical records review. This study used 5 machine learning algorithms to predict the PE: deep neural network (DNN), logistic regression (LR), support vector machine (SVM), decision tree (DT), and random forest (RF). Our model incorporated 18 variables including maternal characteristics, medical history, prenatal laboratory results, and ultrasound results. The area under the receiver operating curve (AUROC), calibration and discrimination were evaluated by cross-validation.

**Results:** Compared with other prediction algorithms, the RF model showed the highest accuracy rate. The AUROC of RF model was 0.86 (95% CI 0.80–0.92), the accuracy was 0.74 (95% CI 0.74–0.75), the precision was 0.82 (95% CI 0.79–0.84), the recall rate was 0.42 (95% CI 0.41–0.44), and Brier score was 0.17 (95% CI 0.17–0.17).

**Conclusion:** The machine learning method in our study automatically identified a set of important predictive features, and produced high predictive performance on the risk of PE from the early pregnancy information.

## Introduction

Preeclampsia (PE) is a multisystem disorder obstetrical syndrome affecting 2%–5% pregnant women and is a main contributor of maternal and perinatal morbidity and mortality worldwide (Poon et al., 2019; Gestational Hypertension and Preeclampsia, 2020; Li et al., 2021). PE is also a high risk factor for the development of cerebrovascular and cardiovascular disease in later life and some chronic disease in later life of offspring (Bellamy et al., 2007). At present, the etiology of PE is not clear, and there are still no effective therapies exist for this disease. To date, the only treatment of PE is confined to the control of hypertension, and the early termination of pregnancy remains the most appropriate treatment (Weitzner et al., 2020). Many studies have shown that pregnant women who are at high risk of developing PE are prescribed low-dose aspirin (50–150 mg/d) before 16 weeks of gestation until 36 weeks of gestation or delivery to minimize the incidence of early-onset PE and fetal growth restriction (FGR) (Roberge et al., 2017; Rolnik et al., 2017; Tan et al., 2018a). Recently, a randomized controlled trial of aspirin in the prevention of PE demonstrated that the incidence of early-onset PE was reduced by 62% when aspirin 150 mg/d was administered to pregnant women at high risk of PE from 11–14 weeks of gestation to 36 weeks of gestation or delivery (Wright et al., 2018). Therefore, it is particularly important to identify high-risk groups of PE during the first trimester. Development of a prediction model to pregnant women may increase the ability to identify those at high risk for PE to facilitate timely prevention intervention and improve maternal and offspring outcomes.

In the past two decades, many researchers have established various prediction models of PE. So far, the most promising joint prediction program includes three parameters: the general conditions of pregnant women, serum biochemical indicators, Doppler ultrasound and mean arterial pressure (MAP). Basic risk of pregnant women, uterine artery pulsation index (PI), MAP, serum pregnancy-associated plasma protein-A (PAPP-A), placental growth factor (PIGF), fetal hemoglobin, and cell-free fetal DNA (cffDNA) have shown important roles in the early prediction of PE (Zeisler et al., 2016; Rolnik et al., 2017; Burton et al., 2019). One multi-center prospective study demonstrated that a competitive risk model was established based on Bayesian rule using maternal factors and combinations of MAP, PI, PIGF and PAPP-A. The results showed that the detection rate of preterm PE was 74.8% and the term PE was 41.3% when the false positive rate was 10% (Tan et al., 2018b). The predictive factors included in the models established by different researchers showed large discrepancies, and most of current studies were from developed countries. Therefore, in order to meet the medical standards of developing countries, a high specificity, high sensitivity and low-cost prediction models of PE is still needed.

With the development of the artificial intelligence world, machine learning (ML) algorithms has been gradually applied in the medical fields. Machine learning is a subset of artificial intelligence that imitates the function of the human brain for data processing (Koteluk et al., 2021). Potential mathematical laws from massive data are discovered and useful information extracted to construct related models by machine learning. In recent years, many valuable results have been achieved in the fields of obstetrics and gynecology (Kawakami et al., 2019; Matsuo et al., 2019; Fung et al., 2020). Sonia Pereira et al. used pregnancy-related factors to predict the appropriate delivery method to determine how to better provide medical services for pregnant women and newborns (Pereira et al., 2015). Signorini et al. (2020) found the best-performing random forest method from 5 machine learning techniques for diagnosing the health of fetuses with intrauterine growth restriction. Therefore, we retrospectively used the data from the hospital’s prenatal diagnosis center to find higher prediction performance from various machine learning methods, such as deep artificial neural network (DNN), decision tree (DT), logistic regression (LR), random forest (RF) and support vector machine (SVM), built a machine learning model for predicting PE during early pregnancy, and evaluated the prediction accuracy of this model in Chinese pregnant women.

## Materials and methods

### Data source

**FIGURE 1:**
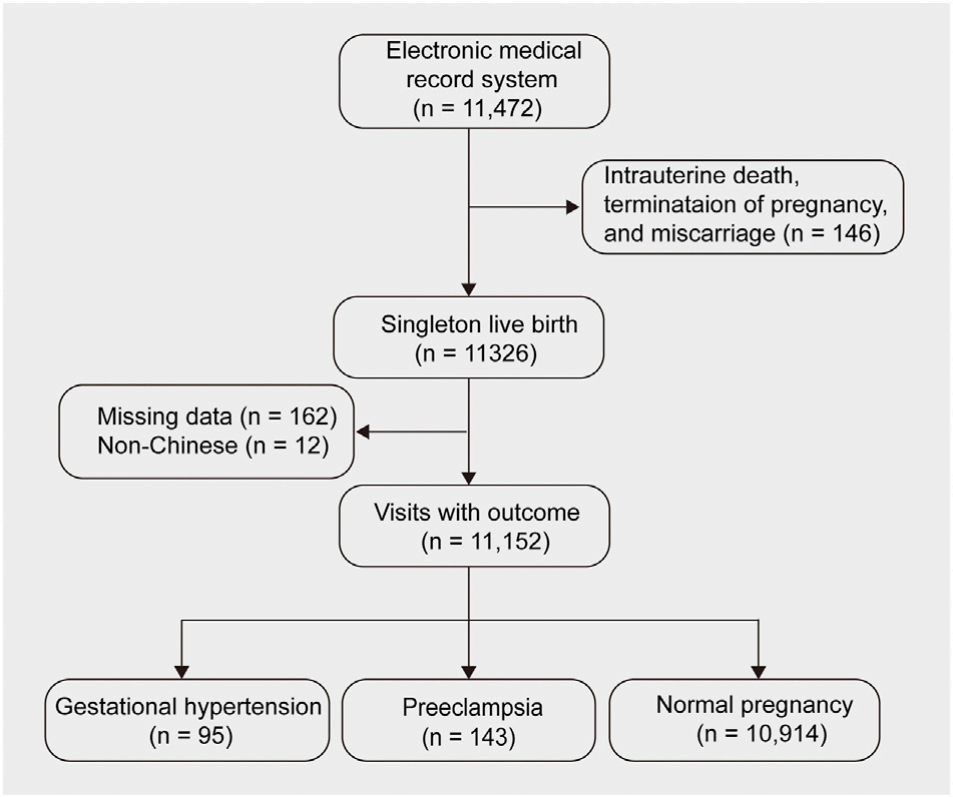
Participant inclusion and exclusion criteria flow diagram.

This was a retrospective cohort study using routinely collected data of aneuploidy screening at The First Affiliate Hospital of Jinan University, at gestational weeks 11^+0^ to 13^+6^ between December 2015 and September 2019. The follow-up data of patients was recorded via medical records, interviews and telephone. A total of 11, 472 singleton pregnancies were collected in this study. Of these, 146 women were excluded from this study due to intrauterine death, termination of pregnancy and miscarriage, 162 patients due to missing data and 12 due to non-Chinese population. A total of 11, 152 pregnant women were included in the final analysis. Among them, 95 were diagnosed with gestational hypertension, 143 with PE, and 10, 914 with normal pregnancy (Figure 1). Antenatal care and evaluations were performed in accordance with the unified strategies of the hospital. The study was approved by the Medical Ethics Committee of First Affiliated Hospital, Jinan University. Given the retrospective study design, informed consent was waived by the institutional review boards.

### Clinical and biochemical data collection

Demographic, laboratory and ultrasonic screening data were collected at the time of fetal aneuploidy screening between 10 and 13 weeks of gestation. The clinical data included age, weight, height, body mass index (BMI), and gestational age at screening. Maternal previous histories of smoking, hypertension, diabetes, FGR, and previous PE as well as obstetrical and social histories and medications prescribed during pregnancy were also recorded. The data of prenatal screening were also collected: β-HCG and pregnancy-associated plasma protein A (PAPP-A). The sonication parameters included crown-rump length (CRL), transparent layer thickness and uterine arteries pulsatility index (UtA-PI). Pregnancy outcomes and complications were taken from the hospital medical records.

### Study outcome

The primary outcome was the occurrence of PE defined as high blood pressure associated with proteinuria. Hypertension was defined as systolic blood pressure (SBP) ≥ 140 mm Hg and diastolic blood pressure (DBP) ≥ 90 mm Hg. Proteinuria was defined as occurrence of one of the following: random urine dipstick results of at least 1 + on two occasions, proteinuria ≥300 mg/24 h, urine protein/creatinine ratio of 30 mg/mmol or any other new-onset sign of PE associated organ dysfunction in the absence of proteinuria (Naseem et al., 2020).

### Selection of prediction model variables

We extracted clinical variables, including demographic characteristics (age, height, weight, smoking history), parity, method of conception, previous diagnosis of hypertension, diabetes, systemic lupus erythematosus (SLE) or antiphospholipid syndrome (APS), the history of GDM or PE, MAP, β-HCG, PAPP-A, and the pulsation index of the bilateral uterine arteries. These predictive indicators were chosen because of strong prior evidence of their association with PE and ease of measuring in clinical practice. In order to address the imbalanced dataset used in this study, the synthetic minority over-sampling technique (SMOTE) was used to deal with imbalanced data. Therefore, smoking history, previous SLE and APS history were not included in the prediction model because the number of cases in the PE group was 0.

### Primary analysis

The clinical history of patient included maternal characteristics, obstetric history, laboratory results, and ultrasound measurement value were collected at 11–13 + 6 weeks of gestation. Categorical variables were recoded numerically before analysis. A total of 11,057 cases were included in the final analysis, of which 143 were in the PE group and 10,914 in the control group. To avoid overfitting and to generalize the models, we used a 10-fold cross-validation. Since the number of samples required for 10-fold cross-validation is a multiple of 10, 7 cases in the control group were randomly removed, and 11,050 cases were finally entered into the model. Stratified random sampling was used to split the data set into 10 sets, and 9 of the 10 sets were used to train the models while the remaining one was used as the testing set. Data was partitioned into a training set to tune algorithms parameters and a test set for evaluation. Through the use of a large number of data sets and evaluation different learning techniques, it has been shown that the 10-fold cross-validation was an appropriate choice to obtain the best error estimate, and there were some theoretical foundations to prove it. In the cross-validation, we repeated each of the 10-fold cross-validation 10 times and reported the average accuracy of the ten 10-fold cross-validation trials.

In our study, PE patients accounted for only 1.3% of the entire sample, while non-PE patients accounted for 98.7%. The difference between these two categories was quite large, which may result in a decrease in the accuracy of the classifier’s predictions. In most cases, real-world data is unbalanced in many applications, such as fraud detection, disease epidemics, credit scoring, or medical diagnosis. Therefore, many well-known methods have been developed and used in machine learning to solve this problem to improve the performance of predictive models (Poolsawad et al., 2014). In our study, the SMOTE algorithm was employed to balance the samples. SMOTE is an oversampling strategy that generates synthetic samples based on feature space similarities between existing minority class examples (Idakwo et al., 2020). Finally, after we split the dataset into training and validation set, we applied SMOTE technology to balance the training datasets, and then standardized the data into the model for training and evaluation.

Five methods were used for prediction model development and compared including LR, DT, SVM, RF, and DNN. LR, DT, SVM, and RF are traditional machine learning models with strong prediction and classification performance. In addition, we also used DNN based on deep learning algorithms to build the model. DNN includes multiple hidden layers to approach the real world with fewer model parameters, faster convergence speed and higher fitting accuracy. For LR, the alpha parameter that defines the strength of regularization term was set to 0.1. For DT, the number of decision trees was set to 100. For SVM, the kernel function was linear. The number of classifiers in RF was 500, the maximum depth of the decision tree was 5, and the number of parallel jobs in the program was -1. The remaining parameters used the default values in the Scikit-learn library.

Each model’s performance was evaluated and compared using the test data set. Finally, the 95% confidence interval was obtained according to the obtained evaluation index. The area under the receiver operating characteristic curve (AUROC) was used to evaluate the model’s ability of discrimination. Calibration was evaluated by the slope, intercept, and Brier score of the calibration curve. Finally, we also reported the accuracy, precision, recall, F1 score and 95% confidence interval of these five algorithms.

### Comparison to previous studies

We also compared the predictive performance of our best model with the results of research over the past five years. We searched PubMed for studies that have developed and/or validated clinical prediction models since 2017. The model must meet the following criteria (Moons et al., 2019): 1) population: for the Chinese pregnant population; 2) index: multivariate clinical prediction model using demographics and clinical predictors; 3) comparator: the best model in this study; 4) outcome: PE does not distinguish between early onset and late onset, with or without FGR; 5) timing: during pregnancy, until onset or before delivery; 6) setting: administration in medical institutions above the second level. These studies need to report the evaluation indicators of predictive efficacy, the sample size of the case group and the control group, the relevant indicators included in the model, and whether the model is validated. This is part of the quality assessment that we follow from the Predictive Model Deviation Risk Assessment Tool (PROBAST) (Wolff et al., 2019). All authors independently evaluated these criteria in the order described. If there are differences between the authors, they can be resolved through discussion.

### Statistical analysis

Patient characteristics were presented as mean ± standard deviation (SD) or median with interquartile range (IQ) for continuous variables, and categorical variables by using frequencies (percentage). The independent sample *t* test or the Mann-Whitney *U* test was used for the continuous variables and the chi-square test or Fisher’s exact probability tests for categorical variables. All statistical analyses were done using SPSS (version 24.0, IBM Corp. Armonk, NY, United States) and the Python software (Version 3.7.0). All statistical tests were two-tailed and *p* < 0.05 was considered statistically significant.

## Results

### Clinical characteristics

The clinical characteristics of study subjects obtained at early second trimester were shown in Table 1. Of the 11, 152 singleton pregnant women included in this study, 143 (1.28%) pregnant women were diagnosed with PE. Comparison of basic indicators of patients in the two groups: there was statistically significant difference in maternal age, body mass index (BMI), previous PE, chronic hypertension, MAP, uterine artery pulsatility index, and serological indicators between the two groups.

**TABLE 1:** Demographic and clinical characteristics of the study population.

Variables	Control (*n* = 10,914)	Cases (*n* = 143)	p Value
Maternal age, y	29 (27–33)	31 (28–36)	<0.001
Weight, kg	53 (48–58)	57 (53–65)	<0.001
Height, cm	160 (156–163)	159 (156–161)	0.037
BMI, kg/m^2^	20.58 (19.02–22.52)	22.88 (20.60–25.30)	<0.001
Gestational age at screening, d	87 (84–90)	87 (84–90)	0.525
Method of conception, n (%)			0.376
Natural	10,684 (97.89)	142 (99.30)	
Assisted	230 (2.11)	1 (0.70)	
Smoking, n (%)	8 (0.07)	0 (0)	1.0
Chronic hypertension, n (%)	15 (0.14)	15 (10.49)	<0.001
SLE/APS, n (%)	19 (0.17)	0 (0)	1.0
The history of GDM, n (%)	299 (2.74)	5 (3.50)	0.598
The history of DM, n (%)	11 (0.10)	2 (1.40)	0.012
The history of FGR, n (%)	149 (1.37)	5 (3.50)	0.049
Parity, n (%)			0.933
Nulliparous	5,796 (53.11)	75 (52.45)	
Parous	5,118 (46.89)	68 (47.55)	
The history of PE, n (%)	74 (0.68)	10 (6.99)	<0.001
Mean arterial pressure, mm Hg	82.80 (78.60–87.10)	91.20 (86.45–99.65)	<0.001
Free β-HCG, ng/ml	62.30 (40.30–96.80)	54.70 (38.28–84.85)	0.034
PAPP-A, IU/L	2,890 (1820–4,510)	2,150 (1,190–3,365)	<0.001
Left uterine artery PI	1.82 (1.46–2.24)	2.00 (1.52–2.40)	0.011
Right uterine artery PI	1.75 (1.42–2.16)	1.88 (1.49–2.24)	0.078
Mean uterine artery PI	1.82 (1.51–2.15)	1.91 (1.56–2.25)	0.022

Data are presented as media (interquartile range) unless indicated as n (%).

BMI, body mass index, SLE, systemic lupus erythematosus, APS, antiphospholipid antibody syndrome, GDM, gestational diabetes mellitus, DM, diabetes mellitus, FGR, fetal growth restriction, PE, preeclampsia. PI, pulse index.

### Model performance

**FIGURE 2:**
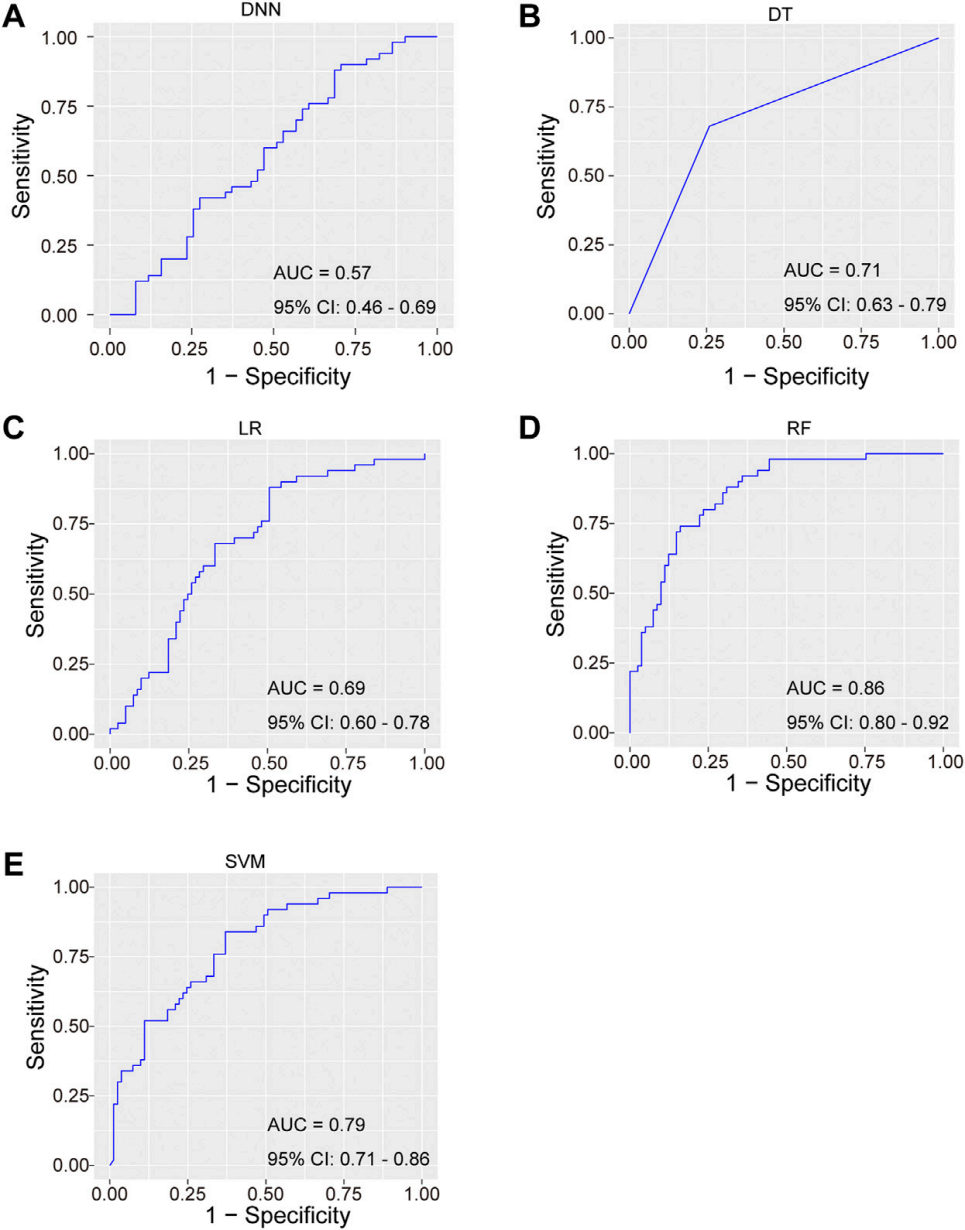
Receiver operating characteristics (ROC) curves for the five machine learning models. **(A)** DNN; **(B)** DT; **(C)** LR; **(D)** RF; **(E)** SVM.

Tables 2, 3 compared the main characteristics of the five models. The RF model showed higher diagnostic performance than the other machine learning models. The AUROC of RF model was 0.86 (95% CI 0.80–0.92), the accuracy was 0.74 (95% CI 0.74–0.75), the precision was 0.82 (95% CI 0.79–0.84), the recall rate was 0.42 (95% CI 0.41–0.44), and F1 score was 0.56 (95% CI 0.54–0.57). The result suggest that the RF model has relatively better negative predictive value. Followed by DNN, LR, SVM, and DT, their AUROCs were 0.57 (95% CI 0.46–0.69), 0.69 (95% CI 0.60–0.78), 0.79 (95% CI 0.71–0.86), and 0.71 (95% CI 0.63–0.79), respectively (Figure 2). Meanwhile, the calibration curve between the actual probability and the predicted probability of the RF model showed that the Brier score was 0.17 (95% CI 0.17–0.17), the slope was 0.92 (95% CI 0.87–0.96), and the intercept was 0.20 (95% CI 0.18–0.21). These results indicated that the prediction model can accurately predict the occurrence of PE and the model was useful in clinical work.

**TABLE 2:** Discrimination tests of five machine learning models for predicting preeclampsia.

Algorithm	Discrimination tests				
	AUROC (95% CI)	Prec. (95% CI)	Accuracy (95% CI)	Recall (95% CI)	F1-score (95% CI)
DNN	0.57 (0.46, 0.69)	0.43 (0.40, 0.46)	0.60 (0.57, 0.64)	0.58 (0.54, 0.62)	0.49 (0.46, 0.53)
LR	0.69 (0.60, 0.78)	0.52 (0.51, 0.53)	0.64 (0.63, 0.65)	0.60 (0.58, 0.62)	0.56 (0.54, 0.57)
SVM	0.79 (0.71, 0.86)	0.56 (0.55, 0.57)	0.68 (0.67, 0.69)	0.77 (0.76, 0.79)	0.65 (0.64, 0.66)
DT	0.71 (0.63, 0.79)	0.61 (0.60, 0.62)	0.70 (0.70, 0.71)	0.62 (0.60, 0.64)	0.61 (0.60, 0.63)
RF	0.86 (0.80, 0.92)	0.82 (0.79, 0.84)	0.74 (0.74, 0.75)	0.42 (0.41, 0.44)	0.56 (0.54, 0.57)

AUROC, area under the receiver operating characteristic curve; Prec. = precision; DNN, deep neural network; LR, logistic regression; SVM, support vector machine; DT, decision tree; RF, random forest.

**TABLE 3:** Calibration tests of five machine learning models for predicting preeclampsia.

Algorithm	Calibration		
	Brier score (95% CI)	Slope (95% CI)	Intercept (95% CI)
DNN	0.25 (0.23, 0.26)	0.17 (–0.00, 0.35)	0.20 (0.12, 0.29)
LR	0.25 (0.24, 0.26)	0.44 (0.39, 0.50)	0.16 (0.14, 0.18)
SVM	0.24 (0.23, 0.25)	0.48 (0.40, 0.55)	0.16 (0.11, 0.21)
DT	0.22 (0.21, 0.23)	0.60 (0.57, 0.63)	0.18 (0.17, 0.19)
RF	0.17 (0.17, 0.17)	0.92 (0.87, 0.96)	0.20 (0.18, 0.21)

DNN, deep neural network; LR, logistic regression; SVM, support vector machine; DT, decision tree; RF, random forest.

### Comparison to previous studies

In the past five years, we have found 286 records from PubMed with the keyword “preeclampsia prediction and China”, of which 5 studies were eligible and can be compared with our RF model. Compared with most previous models, the RF model has the better prediction performance, showing higher prediction accuracy and calibration (Table 4). This means that the prediction model established by machine learning can improve the precision and accuracy of prediction.

**TABLE 4:** Predictive performances shown by DNN models in this study compared to those from previous studies.

Source	Predictive performance		
	AUROC (95% CI)	Prec. (95% CI)	Sens. (95% CI)
RF in this study	0.86 (0.80, 0.92)	0.82 (0.79, 0.84)	0.42 (0.41, 0.44)
Li et al. (2021)	0.96	0.45	0.79
Zhang et al. (2021)	0.95 (0.91, 0.99)	NA	0.88
Yue et al. (2021)	0.83 (0.79, 0.88)	NA	NA
Hou et al. (2020)	0.88 (0.84, 0.92)	NA	0.90
Quan et al. (2018)	NA	NA	0.91

AUROC, area under the receiver operating characteristic curve; Prec, precision; Sens, sensitivity; DNN, deep artificial neural network; NA, not available.

## Discussion

In this study, we successfully developed a good prediction model for PE by using various machine learning algorithm. Compared with other prediction algorithms, the RF mode showed the highest accuracy rate. More importantly, these models obtained high predictive power using prenatal screening data readily available to the obstetrician at the time of early pregnancy.

PE is a major cause of maternal and fetal morbidity and mortality, which the development of prediction models has always been a hot topic in the field of obstetrics. At present, scholars at home and abroad have established a variety of predictive models for PE. Common predictive factors include maternal characteristics, genetic indicators, Doppler indicators, and biochemical indicators. However, the current prediction models have disadvantages such as difficult access to indicators and lack of validation of the model, which limit their clinical application. Therefore, novel statistic approaches are urgently needed to establish an early predictive model of PE that is suitable for the real maternity examination situation of domestic maternal and child health care, and pay attention to the collection of indicators in line with the real clinical situation.

Random forests is a Bagging ensemble learning algorithm based on decision tree proposed by Breiman (2001). Because of its high accuracy, fast training speed and effective prevention of overfitting, random forest has become a popular machine learning method in clinical research (Signorini et al., 2020; Wang et al., 2020; Schmidt et al., 2022). We built prediction models by five machine learning methods using the data of antenatal screening in early pregnancy. All these predictors were routinely available, quickly measured and relatively inexpensive. Besides, these predictors have been previously identified as risk factors for PE. Age, BMI, diabetes mellitus, and chronic hypertension were independent predictors of PE and used in the ACOG and NICE guidelines (National Collaborating Centre for and Children’s, 2010; Alldred et al., 2017; Rocha et al., 2017). However, judging the risk of PE based on only high-risk factors may have some drawbacks. One is that the screening rate is low; the other is that most pregnant women with high-risk factors do not actually have PE, which resulted in false positive rate being too high and unnecessary interventions. In recent years, a large number of studies have found that the prediction efficiency of complex models combined with auxiliary inspections is significantly higher than that of simple models (Wright et al., 2012; O'Gorman et al., 2016; Jhee et al., 2019; Antwi et al., 2020; Serra et al., 2020; Wright et al., 2020). At present, most studies used the multiple logistic regression algorithm to predict the risk of early-onset PE, or used the Bayesian principle to calculate the prior risk with a simple multiple logistic regression model, and then use the likelihood ratio in combination with special inspections to calculate the posterior risk of PE. This algorithm usually needs to use different formulas to evaluate the risk of PE and the included prediction indicators are often different. In recent years, more and more studies have found that the pathogenesis of early-onset PE and late-onset PE cannot be clearly distinguished. Some scholars have begun to explore modeling algorithms other than the logistic regression model. Some studies have established a competitive risk model to calculate the time relationship between the gestational week of PE and the gestational week of delivery (Wright et al., 2015; Wright et al., 2020). The British FMF established and continuously improved the competitive risk model to predict PE and the model can be openly used on the foundation website (https://fetalmedicine.org/calculator/preeclampsia) (Akolekar et al., 2013; O'Gorman et al., 2016). Al-Rubaie et al. conducted a systematic review of prediction models of PE and established a prediction model. The prediction performance of the model varies greatly. The area under the receiver operating curve (AUC) fluctuates between 0.64 and 0.96, the sensitivity 29%–100%, and the specificity 26%–96%, but all prediction models lack sufficient external verification (Brunelli and Prefumo, 2015; Al-Rubaie et al., 2016). Recently, several machine learning strategies have been developed with the use of second-trimester data to predict late-onset PE (Jhee et al., 2019). In our research, we combined maternal medical history and prenatal screening laboratory indicators (PAPP-A and β-HCG) with ultrasound indicators (uterine artery PI) to establish a new predictive model through machine learning. Our model provided a plausible predictive tool for identifying the high-risk pregnant women among Chinese population.

One major limitation of our study is that our models have not been validated using external data sets. However, our inclusions criteria were accurately defined to facilitate future external verification. Perhaps due to the true difference in the incidence of PE, the prevalence of PE in Southeast Asia is less than 2% (Ilekis et al., 2007; Leung et al., 2008). The number of pregnant women in the PE group and the non-PE group was very different, and the sample was also not balanced. Although a SMOTE algorithm was used to balance the data, some bias may still exist between the two groups and it was easy to produce the problem of marginalization of the distribution, which blurred the boundary between the two types of samples and increased the difficulty of classification by the classification algorithm. In the follow-up external verification, we will explore more suitable algorithms to solve the problem of data imbalance. Finally, our data was single-center, which might hamper generalizing its findings.

Our study demonstrated that machine learning was a promising diagnostic tool for PE. With higher performance, machine learning can predict PE prospectively. Based on the patient’s clinical history and prenatal screening results, the predictive model calculates the score of each patient to assess the chance of PE using RF. This makes it possible to identify high-risk patients and start treatment with low-dose aspirin.

## Data Availability

The raw data supporting the conclusion of this article will be made available by the authors, without undue reservation.
