# Supplementary material for: Development of a prediction model on preeclampsia using machine learning-based method: a retrospective cohort study in China
Source: Front Physiol. 2022 Aug 12;13:896969. doi: 10.3389/fphys.2022.896969 (PMC9413067; doi:10.3389/fphys.2022.896969)
Supplement: Supplementary file 1 [file DataSheet1.docx]

Supplement Table 1. Discrimination tests of five machine learning models for predicting preeclampsia with 1:1 cases in normal group and PE group.

| Algorithm | Discrimination tests | | | | |
| --- | --- | --- | --- | --- | --- |
|  | AUROC (95%CI) | Prec. (95%CI) | Accuracy (95%CI) | Recall (95%CI) | F1-Score (95%CI) |
| DNN | 0.91 (0.91, 0.92) | 0.83 (0.82, 0.83) | 0.83 (0.82, 0.84) | 0.84 (0.82, 0.85) | 0.83 (0.83, 0.84) |
| LR | 0.88 (0.85, 0.91) | 0.79 (0.77, 0.82) | 0.80 (0.77, 0.84) | 0.82 (0.75, 0.88) | 0.80 (0.76, 0.85) |
| SVM | 0.87(0.86, 0.91) | 0.79 (0.76, 0.82) | 0.81 (0.76, 0.85) | 0.84 (0.77, 0.91) | 0.81 (0.77, 0.86) |
| DT | 0.83 (0.82, 0.85) | 0.71 (0.68, 0.74) | 0.74 (0.70, 0.78) | 0.82 (0.76, 0.88) | 0.76 (0.72, 0.80) |
| RF | 0.87 (0.84, 0.91) | 0.81 (0.78, 0.83) | 0.79 (0.74, 0.84) | 0.76 (0.67, 0.85) | 0.78 (0.72, 0.84) |

Supplement Table 2. Discrimination tests of five machine learning models for predicting preeclampsia with 1.5:1 cases in normal group and PE group.

| Algorithm | Discrimination tests | | | | |
| --- | --- | --- | --- | --- | --- |
|  | AUROC (95%CI) | Prec. (95%CI) | Accuracy (95%CI) | Recall (95%CI) | F1-Score (95%CI) |
| DNN | 0.91 (0.90, 0.91) | 0.80 (0.79, 0.80) | 0.83 (0.83, 0.84) | 0.79 (0.77, 0.80) | 0.79 (0.78, 0.80) |
| LR | 0.88 (0.85, 0.91) | 0.76 (0.73, 0.79) | 0.80 (0.76, 0.83) | 0.72 (0.65, 0.80) | 0.80 (0.76, 0.85) |
| SVM | 0.88 (0.85, 0.91) | 0.75 (0.72, 0.78) | 0.80 (0.76, 0.83) | 0.74 (0.67, 0.82) | 0.74 (0.69, 0.79) |
| DT | 0.74 (0.70, 0.78) | 0.63 (0.59, 0.66) | 0.73 (0.69, 0.77) | 0.79 (0.71, 0.87) | 0.70 (0.65, 0.75) |
| RF | 0.87 (0.83, 0.90) | 0.77 (0.73, 0.80) | 0.79 (0.75, 0.82) | 0.66 (0.59, 0.73) | 0.71 (0.66, 0.76) |

Supplement Table 3. Discrimination tests of five machine learning models for predicting preeclampsia with 2:1 cases in normal group and PE group.

| Algorithm | Discrimination tests | | | | |
| --- | --- | --- | --- | --- | --- |
|  | AUROC (95%CI) | Prec. (95%CI) | Accuracy (95%CI) | Recall (95%CI) | F1-Score (95%CI) |
| DNN | 0.91 (0.90, 0.91) | 0.79 (0.78, 0.79) | 0.85 (0.84, 0.85) | 0.74 (0.71, 0.76) | 0.76 (0.75, 0.78) |
| LR | 0.88 (0.85, 0.91) | 0.73 (0.69, 0.77) | 0.80 (0.77, 0.84) | 0.65 (0.57, 0.73) | 0.69 (0.62, 0.75) |
| SVM | 0.88 (0.85, 0.91) | 0.72 (0.68, 0.77) | 0.81 (0.77, 0.84) | 0.66 (0.58, 0.75) | 0.69 (0.63, 0.75) |
| DT | 0.73 (0.68, 0.77) | 0.60 (0.54, 0.66) | 0.74 (0.70, 0.77) | 0.69 (0.58, 0.80) | 0.63 (0.58, 0.69) |
| RF | 0.87 (0.83, 0.90) | 0.76 (0.72, 0.81) | 0.80 (0.77, 0.83) | 0.57 (0.50, 0.63) | 0.65 (0.59, 0.71) |
